# Z-Drugs Prescribing in a Community Mental Health Team Setting in Helensburgh, Scotland: A Cross-Sectional Survey

**DOI:** 10.1192/bjo.2025.10093

**Published:** 2025-06-20

**Authors:** Felix Kauye, Amanthi Wijesundara, Dalitso Mwandumba

**Affiliations:** ^1^NHS Greater Glasgow and Clyde, Glasgow, United Kingdom; ^2^University of Glasgow, Glasgow, United Kingdom

## Abstract

**Aims:** Managing insomnia is a common challenge for psychiatrists and their patients. A real-world cohort study on first-line treatment patterns in 265,382 patients with insomnia found that 42.4% of that group were prescribed hypnotic medications. Among those, first prescriptions were most frequently a Z-drug with 35.8% of all patients on these medications. Z-drugs include zolpidem, zopiclone, eszopiclone and zaleplon. We aimed to assess the prevalence and associated factors of Z-drug prescribing in a Community Mental Health Team (CMHT) setting in Helensburgh, Scotland.

**Methods:** A cross-sectional survey was conducted to examine the prescribing of Z-drugs in a cohort of 412 patients attending the Helensburgh CMHT outpatient clinics between May and August 2021. Data on who was on Z-drugs was extracted from General Practitioner records on Clinical Portal. Once the list of who was on Z-drugs was compiled, associated factors were extracted from their Electronic Medical Information System (EMIS) records. Analyses were done to compare the characteristics of those on Z-drugs versus those not on Z-drugs. Continuous factors were found to be approximately normally distributed and were compared between groups using the unpaired t-test. The Chi-square test was used to compare categorical variables between groups.

**Results:** Of the 412 patients in the dataset, 30 (7%) were on Z-drugs. Zopiclone and zolpidem were the only Z-drugs prescribed with rates of 86.7% and 13.3% respectively. The analysis results suggested that age, sex and the number of psychiatric medications varied significantly between those on Z-drugs compared with those not on Z-drugs. However, the primary diagnosis of the groups was not significantly different (p=0.63). The group on Z-drugs had a higher proportion of females (77%) than the group not on Z-drugs (57%) (p=0.04). Z-drug users were older, with mean age of 51.5±14.0 years, compared with a mean age of 42.0±14.7 years in the group not on Z-drugs (p=<0.001). The number of psychiatric medications was higher in Z-drug users i.e. 3.4±1.0 compared with 1.9±1.2 for those not on Z-drugs (p=<0.001).

**Conclusion:** The prevalence of Z-drug prescriptions can vary significantly across different countries and regions, which makes it challenging to generalize. Rebound insomnia and withdrawal symptoms occur infrequently upon discontinuation, however it may be less common and milder than those seen upon discontinuation of benzodiazepines. Z-drugs have known side effects including headaches, dizziness, anterograde amnesia, confusion, and hallucinations, and it is important to monitor their prescribing and associated factors in different settings including secondary care.

